# Simulation and Experimental Study on the Precision Molding of Irregular Vehicle Glass Components

**DOI:** 10.3390/mi14101974

**Published:** 2023-10-23

**Authors:** Zhijun Chen, Shunchang Hu, Shengfei Zhang, Qingdong Zhang, Zhen Zhang, Wuyi Ming

**Affiliations:** 1 School of Mechanical Engineering, University of Science and Technology Beijing, Beijing 100083, China; czj5332@gmail.com (Z.C.); zl86860908@163.com (Q.Z.); 2 Mechanical and Electrical Engineering Institute, Zhengzhou University of Light Industry, Zhengzhou 450002, China; hushunchang2022@gmail.com; 3 School of Aerospace Engineering, Huazhong University of Science and Technology, Wuhan 430074, China; zzhen@hust.edu.cn; 4 Guangdong Provincial Key Laboratory of Digital Manufacturing Equipment, Guangdong HUST Industrial Technology Research Institute, Dongguan 523808, China

**Keywords:** large irregular glass components, molding, optimization, energy consumption

## Abstract

The high level of stress and dimension deviation induced by glass molding are the main causes of the low yield rate of large, irregular glass components on vehicles. To solve this issue, a numerical model of large glass component molding was established in this study, which aimed to analyze the dominant factors of molding quality and achieve a synergistic balance between quality characteristics and energy consumption. The results show that molding temperature is the dominant factor affecting the energy consumption and residual stress, and the molding pressure is the main factor affecting the dimension deviation. Furthermore, the NSGA-II optimization algorithm was used to optimize the maximum residual stress, dimension deviation, and energy consumption with the numerical results. The combination of a heating rate of 1.95 °C/s, holding time of 158 s, molding temperature of 570 °C, molding pressure of 34 MPa, and cooling rate of 1.15 °C/s was determined to be the optimized scheme. The predictive error of the numerical result, based on the optimized scheme, was experimentally verified to be less than 20%. It proved the accuracy of the model in this study. These results can provide guidance for the subsequent precision molding of large, irregular glass components.

## 1. Introduction

**Figure 1:**
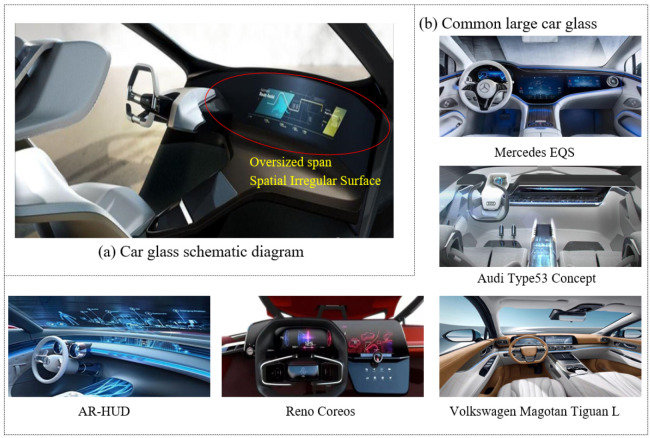
Common 3D glass components of vehicles.

Today, with the rapid development of vehicles, communications, electronic products, and other industries, 3D curved glass offers a good visual experience to users due to the surface treatment of the arc in the middle or edge positions as it is in line with the arc of the human retina [1]. A new glass machining process, molding technology [2], has been widely applied in the 3D glass manufacturing field as it has the advantages of high molding accuracy and low equipment cost [3,4,5,6]. During molding, the glass blank undergoes three procedures, as follows: heating and softening, pressure loading, and cooling. During this process, improper molding parameters may cause issues of uneven glass flow (resulting in small defects) [7], geometric dimension deviation, high stress, and other problems associated with glass products. The glass components of vehicles are usually large in size and have irregular structures (as shown in Figure 1); therefore, their molding quality problems are more prominent. In addition, molding is a high-energy process, and high-quality glass components are usually manufactured at the expense of production costs [8]. Therefore, establishing a prediction model for the precision molding of large glass components, studying molding quality and the tendency to produce large, irregular glass components for vehicles during molding, and balancing the relationship between glass molding quality and energy consumption are urgent problems to be addressed [9].

Many studies have been conducted on glass molding [10]. For example, Yan et al. [11] proposed a new two-step hot pressing method, and they established a glass heat transfer model by considering the nonlinear characteristic of the material’s thermal expansion and the specific interaction between heat, thermal conductivity, and the temperature of glass materials. The improved Newtonian fluid model was intended to describe the flow of glass materials at high temperatures, and the load fluctuation during extrusion was accurately predicted. Tao et al. [12] found that the residual stress is significantly affected by the thermal expansion coefficient and the heat capacity of the glass material. Sarhadi et al. [13] quantified the friction coefficient and viscoelastic properties of the glass/mold interface via simulations and experimental testing, and they successfully predicted the final dimensions and residual stress distribution of the molded lens. Schott [14] and Fotheringham [15] found that geometric accuracy affects the refractive index of glass components, and the curvature of the glass components may deviate with changes in temperature and structure [16]. Further, Balajee et al. [17] studied the influence factors of the dimension deviation of glass components, including molding temperature, molding rate, and mold parameters. By analyzing the geometric deviation of glass, Dambon et al. [18] adopted the finite element method, geometric compensation method, and other related “high-tech” methods that can assist in molding and manufacturing.

The machining quality of the product depends on the process parameters [19,20]. It is important to model accurate prediction models and optimize the process parameters to improve product quality [21]. In recent decades, many studies have focused on the correlation and optimization of machine quality and process parameters [22,23,24,25,26,27]. For example, He et al. [27] conducted a systematic study on the molding of the 3D curved screens of smartphones, and a multi-objective optimization method based on the genetic algorithm (GA) was applied to efficiently solve the optimization problem of glass product quality. Li et al. [26] established a theoretical model of grinding force by taking the comprehensive influence of strain rate, random distribution of abrasive radii, and “elastic-to-plastic” transition depth into account; the key force model parameters were determined using a GA trained based on the experimental data. Zhang et al. [24] developed a mixed prediction model of residual stress using fuzzy theory and a GA. Chen et al. [25] predicted and optimized the residual stress of Mg-Li alloy milling based on the GRA-BP-NSGA-III model.

In summary, the above studies have verified the influence of glass material properties and molding parameters on glass molding quality and realized the optimization of the molding process [28,29]. However, these studies on the molding technology for glass components mainly focus on small glass components, and the research on large glass components is limited. For example, the size of the 3D curved screen on a smartphone is 148 × 73 × 0.35 mm, the fingerprint lock glass panel is 348 × 66.6 × 2 mm, and the mobile display obtuse glass component is *Φ*14 × 7 mm, as shown in Table 1. However, the glass component of vehicles studied in this study is a large, irregular ultra-thin curved glass component with a size of 1454 × 161 × 2 mm and an aspect ratio of 9.03. Large and irregular structures of glass components greatly increase the difficulties during glass molding, easily producing large residual stress, large dimension deviation, and other issues, resulting in the problems of low yield rate and low productivity of the components. In order to improve the product quality of large glass components and balance the relationship between product quality and production efficiency, a numerical model of glass molding for large instrument panels on vehicles was established to predict the molding process and quality of glass components. The molding quality and energy consumption of glass components were analyzed and optimized based on experimental data with multi-variable factors. Through experimental verification, multi-objective cooperative optimization between glass product quality and energy efficiency was realized.

## 2. Numerical Simulation and Analysis

### 2.1. Numerical Modeling

MSC. Marc is a powerful thermo-mechanical coupling simulation software with high-precision convergence solution technology, which can achieve the thermal analysis of steady and transient states and can effectively simulate complex nonlinear problems. In this study, the molding processes of large glass components of vehicles were simulated based on the software of MSC. Marc 2019. Through real-time observation of the temperature distribution, deformation, and thermal stress of glass blank in multiple stages of heating, molding, and cooling, the molding mechanism of large glass components during molding can be better understood.

(1) Geometric model

**Figure 2:**
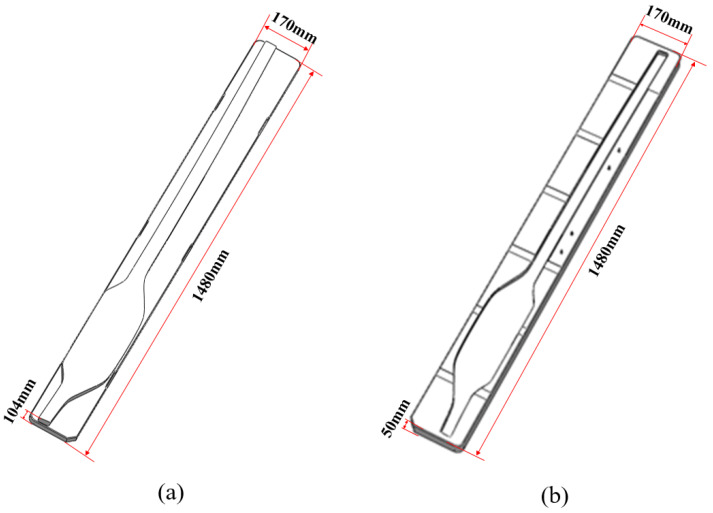
Three-dimensional models for the glass molds of vehicles: (**a**) upper mold; (**b**) lower mold.

**Figure 3:**
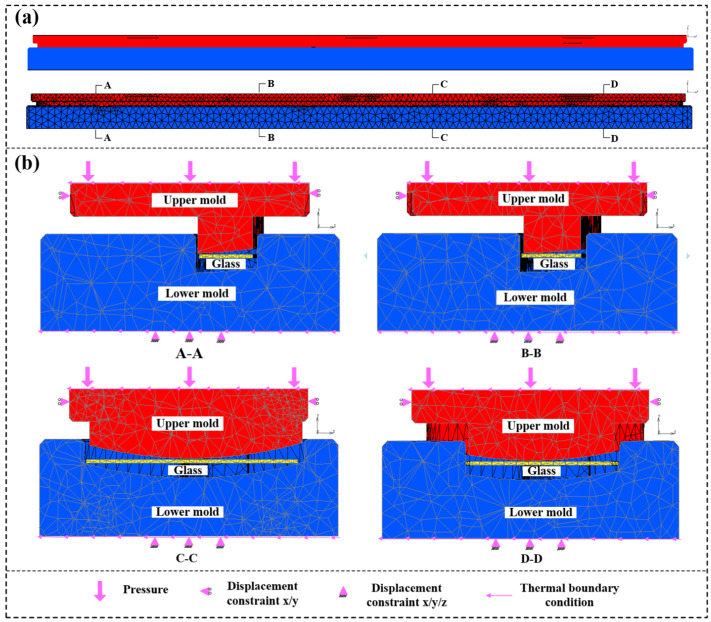
Finite element model of glass and molds: (**a**) overall model; (**b**) sectional views of positions A, B, C, and D.

In this study, a large glass component with a complex asymmetric structure on a vehicle is the research object, and its mold model is shown in Figure 2. The sizes of both the upper and lower molds are 1480 × 170 mm, and the heights of the upper and lower molds are 104 and 50 mm, respectively. In order to accurately describe the shape of the glass component, a solid tetrahedral element was used to divide molds, and mesh refinement was performed on the glass blank and local areas of molds. The final model consists of 116,851 mold elements and 36,986 glass elements, as shown in Figure 3.

(2) Material models

Third-generation Corning glass and graphite were selected as the glass components of vehicles and mold materials, respectively; their thermo-mechanical properties are shown in Table 2. The viscoelastic characteristics of the glass material are shown in Table 3. In order to better describe the characteristics of structural and stress relaxations of glass materials, the glass viscosity change with temperature was predicted with the William–Landel–Ferry model, and the structural relaxation of glass materials with temperature was described with the Narayanswamy model [34,35]. The parameters of the William–Landel–Ferry and Narayanswamy models are shown in Table 4.

(3) Boundary conditions

**Figure 4:**
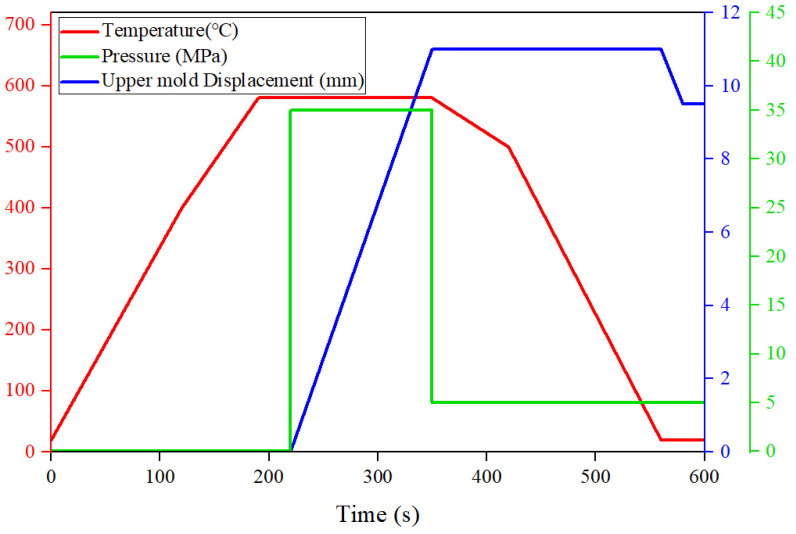
Schematic diagram of boundary conditions.

The initial temperatures of the molds and glass blank were set at 20 °C, the thermal conduction coefficient between the mold and glass was set as 2800 W/m^2^K, and the friction coefficient between the glass materials and mold was set as 0.1. According to the actual situation, boundary conditions and loading conditions of the model were determined, the movements in the *x*, *y,* and *z* directions of the lower mold were restricted, and the movements in the *x* and *y* directions of the upper mold were restricted. At the same time, the upper mold moves in the negative direction of the *z*-axis under a certain pressure. As shown in Figure 4, the temperature control strategy during glass molding is mainly divided into three parts: heating, holding, and cooling. First, the glass blank temperature begins to rise, reaching the expected molding temperature at 240 s. Then, the upper mold moves 9 mm in the negative direction of the *z*-axis under a steady pressure of 35 MPa to achieve the desired molding effect during the holding stage, and the whole step will last for 150 s. The release of internal residual stress can effectively avoid cracking. Relevant studies have shown that the internal stress of glass materials can be alleviated effectively at around 500 °C [27,36]. Therefore, in this study, glass components and molds were slowly cooled to around 500 °C to effectively release the internal stress, followed by rapid cooling to room temperature. In order to ensure the dimensional accuracy of glass components, a load of 450 N was applied to the upper mold during cooling. The loading conditions at each stage are shown in Figure 3.

**Table 4:** Relaxation model parameters of third-generation Corning glass [37].

Stress Relaxation		Structural Relaxation	
Shear Modulus (MPa)	Relaxation Time (s)	Weight Coefficient	Relaxation Time (s)
12,566	0.0689	0.108	3.0
		0.443	0.671
12,615	0.0065	0.166	0.247
		0.161	0.091
4582	0.0001	0.046	0.033
		0.077	0.008

### 2.2. Result Analysis

**Figure 5:**
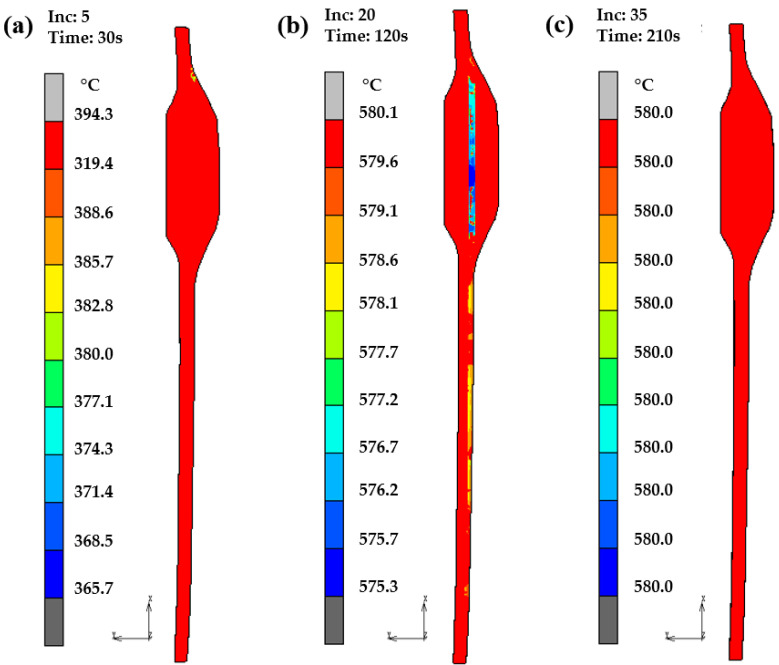
Temperature distribution of glass component of vehicle during heating: (**a**) 30 s; (**b**) 120 s; (**c**) 210 s.

Figure 5 shows the temperature variation during the heating stage. At 30 s, the glass component of a vehicle presents temperature distribution at different positions; there is a temperature difference of 28.6 °C between the middle and edge of the glass component. After 120 s, the temperature difference in the glass component becomes very small, only about 4.8 °C. As heating time increases, as shown in Figure 5c, the temperature distribution gradually becomes uniform, and it eventually reaches stability at 580 °C.

**Figure 6:**
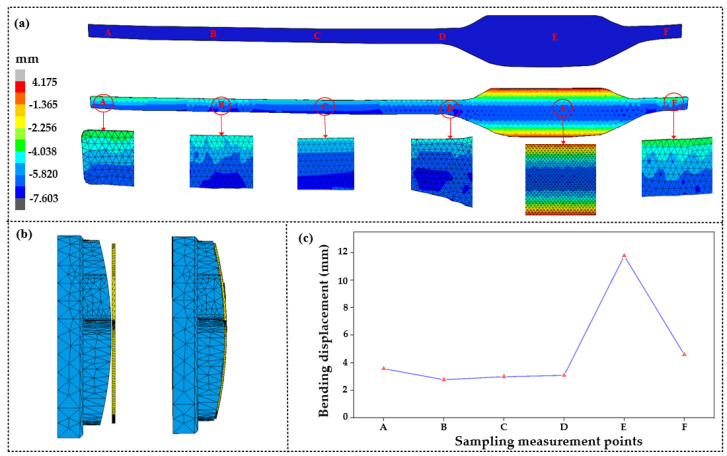
Deformation displacement on the *z*-axis of the glass component after molding: (**a**) deformation displacement at the positions of A–F; (**b**) fit degree; (**c**) displacement of the sampling positions A–F.

As heating proceeds, the temperature of the glass component reaches the expected molding temperature, and the glass begins to deform under the pressure exerted by the upper mold. Figure 6a shows the deformation displacement in the local areas of the glass component during molding, and it is found that the maximum *z*-axis displacement at position *E* is 11.775 mm. The fit degree between the glass and mold in the direction of the *z*-axis was analyzed, and it can be seen that there is no obvious gap between the glass and lower mold in Figure 6b. Figure 6c shows the line displacement of the glass component at positions *A*~*F*. It can be observed that there is a little deformation displacement from position *A* to *D*, but the bending degree of the glass component is large from position *D* to *F*. From the above analysis, it can be concluded that the bending deformation of the glass component mainly occurs at the position of the main cavity. In this study, the fit gap between the glass and lower mold in the middle of the main cavity was selected as the evaluation standard of the dimension deviation.

### 2.3. Scheme Design

In this study, different process conditions, including molding temperature, heating rate, molding pressure, holding time, and cooling rate, were studied to assess the residual stress, dimension deviation, and energy consumption during molding. In order to better explore the influence of various factors on the glass molding process, an orthogonal experimental design based on the molding parameters was implemented. The molding parameters of heating rate (*A*), holding time (*B*), molding temperature (*C*), molding pressure (*D*), and cooling rate (*E*) were determined as the control variables; four levels were designed for each control factor, and an orthogonal array of L_16_ (4^5^) was designed. The level setting standards of the five control factors can be seen in Table 5. Finally, 16 data samples were collected through finite element analysis, as shown in Table 6. Then, the collected sample data were further analyzed for the influence of different control factors on residual stress, dimension deviation, and energy consumption.

## 3. Influence of Molding Parameters on Quality and Energy Consumption

### 3.1. Residual Stress

**Figure 7:**
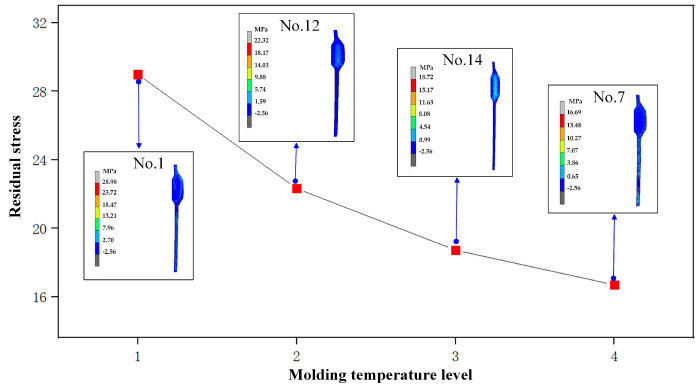
Influence of molding temperature on residual stress.

Through the orthogonal experimental analysis, it could be concluded that molding temperature (*C*), molding pressure (*D*), and cooling rate (*E*) are the main factors affecting the residual stress, as shown in Table 7. In Figure 7, it can be seen that the maximum value of the residual stress has an obvious linear relationship with molding temperature. At 580 °C (No. 7), residual stress reaches the minimum value of 15.10 MPa; as molding temperature decreases, residual stress increases to 28.98 MPa at 550 °C (No. 1). The viscosity of the glass material reduces significantly as temperature increases, and the rheological property improves, which results in a decrease in residual stress.

**Figure 8:**
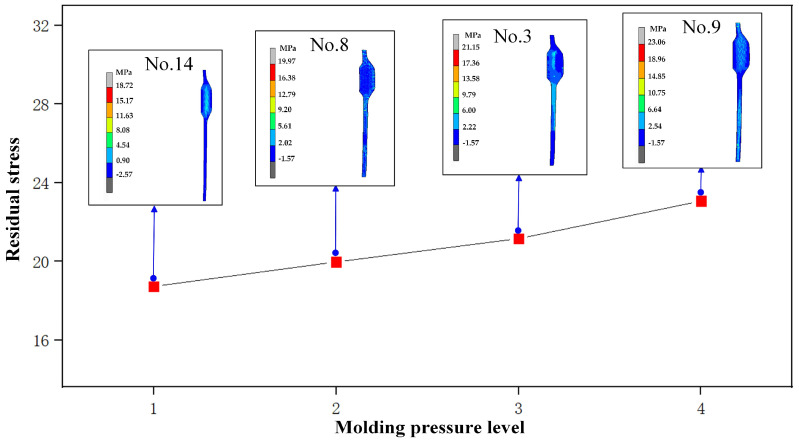
Influence of molding pressure on residual stress.

As shown in Figure 8, the maximum value of the residual stress gradually increases with molding pressure. The value of residual stress is 18.75 MPa at a molding pressure of 20 MPa (No. 14), and it reaches 23.06 MPa when the molding pressure increases to 35 MPa (No. 9). As the molding pressure increases, the strain rate of the glass material will be significantly increased, thus shortening the time required for glass deformation, so that the internal balance of the glass material cannot be effectively maintained, and less stress is released in a short time, resulting in an increase in residual stress.

**Figure 9:**
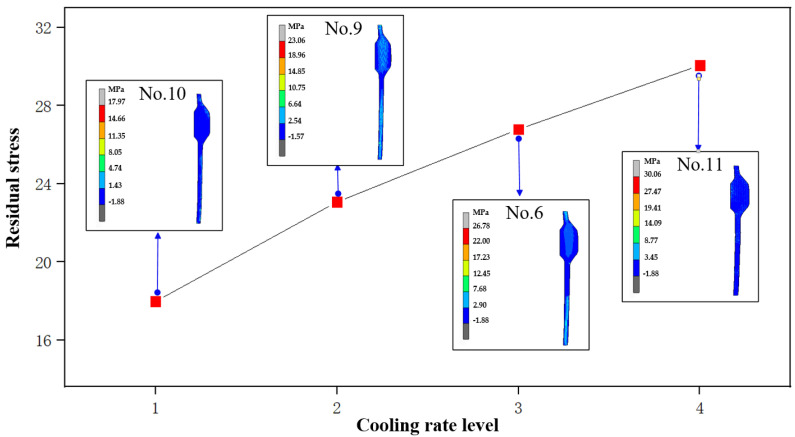
Influence of cooling rate on residual stress.

As can be seen from Figure 9, there is an obvious linear relationship between the cooling rate and residual stress. As the cooling rate increases from 0.75 °C/s (No. 10) to 1.5 °C/s (No. 11), the maximum residual stress value rises from 17.97 MPa to 30.06 MPa. An increase in the cooling rate results in a greater temperature difference within the glass components, leading to an enlarged temperature gradient, which will further increase stress aggravatingly. In addition, accelerating the cooling rate will shorten the cooling time and may cause the internal structure of the glass material to not fully achieve a stable state, thus reducing the ability of stress relief.

### 3.2. Dimension Deviation

**Figure 10:**
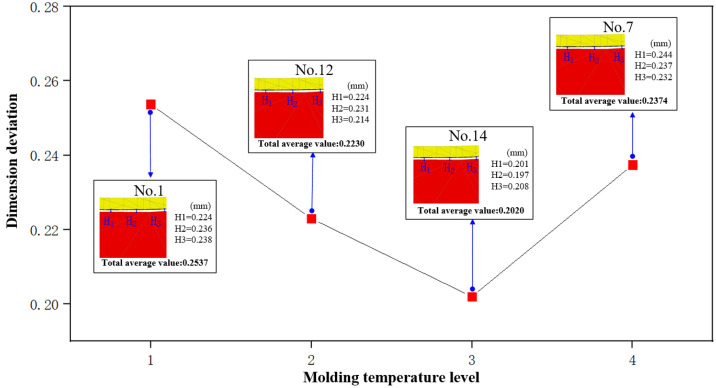
The influence of molding temperature on dimension deviation.

Molding pressure (*D*), cooling rate (*E*), and molding temperature (*C*) are the main factors affecting product dimension deviation, as shown in Table 8. The information from Figure 10 indicates that the dimension deviation alteration follows a pattern of initial reduction followed by enlargement with an increase in molding temperature. As the molding temperature gradually ascends from 550 °C (No. 1) to 570 °C (No. 14), a significant reduction in dimension deviation is observed. At 580 °C (No. 7), dimension deviation tends to increase in reverse. A moderate increase in molding temperature leads to improvements in the flow characteristics of glass materials, contributing to a reduction in dimension deviations. However, excessive temperature may cause the glass materials to undergo deformation beyond the normal range, which results in a corresponding increase in their volumetric expansion rate, further exacerbating their appearance inaccuracies in the subsequent cooling step.

**Figure 11:**
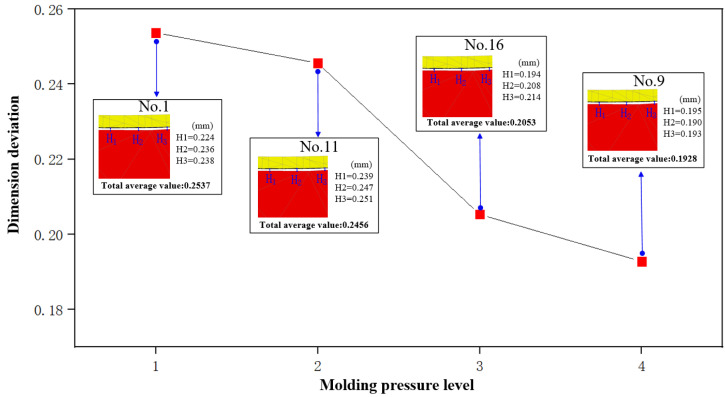
The influence of molding pressure on dimension deviation.

From Figure 11, it is evident that the molding pressure significantly influences the dimension deviation of the molded glass components. At a molding pressure of 20 MPa (No. 1), dimension deviation is recorded at 0.2537 mm. As molding pressure escalates to 35 MPa (No. 9), there is a noticeable reduction in dimension deviation, decreasing from the initial value of 0.2537 mm to 0.1982 mm. Furthermore, with the increase in molding pressure, there is an enhancement in filling efficiency between the glass and molds, effectively reducing dimension deviation.

**Figure 12:**
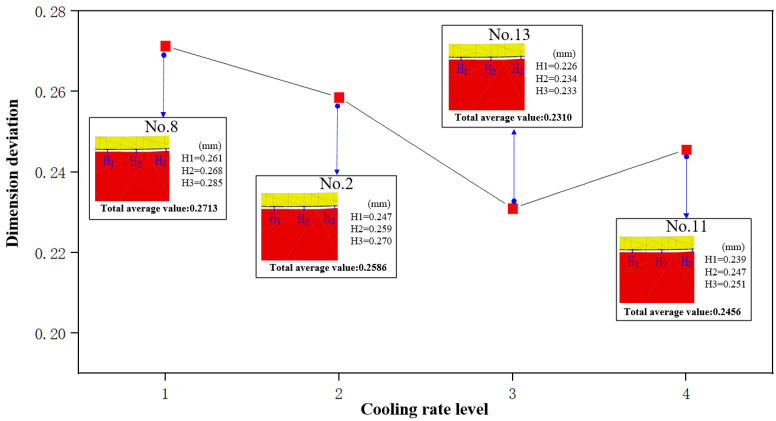
The influence of cooling rate on dimension deviation.

According to Figure 12, we can observe that as the cooling rate increases from 0.75 °C/s (No. 8) to 1.5 °C/s (No. 11), dimension deviation initially decreases and then increases again, ultimately reaching a turning point at 1.25 °C/s. In theory, as the cooling rate increases, there is a corresponding reduction in the required time. This poses a greater challenge for the structure inside glass material to achieve a stable state, resulting in reduced deformation due to material structural relaxation. Consequently, the dimension deviation decreases. However, an excessive cooling rate may also lead to mold deformation, resulting in the deformation of glass components.

### 3.3. Energy Consumption

The calculation of energy consumption in glass molding encompasses not only the heat absorbed by the mold and glass but also the heat consumption by nitrogen [38]. These heat contributions over a production cycle can be expressed using Equations (1) and (2):

(1) $$E_{e}=\lambda (Q_{1}+Q_{2})=\lambda (\sum_{i=1}^{2}c_{i}m_{i}\Delta T+c_{3}vt\rho \Delta T\prime )$$

(2) $$\lambda (T)=2.01+3.1\times 10^{-5}T$$

where:

$E_{e}$—energy consumption (kJ/pcs);

λ—thermal loss coefficient, a function of temperature *T*;

$Q_{1},Q_{2}$—heat consumption of the glass blank and molds, and heat consumption of nitrogen (kJ/pcs);

$c_{1-3}$—specific heat capacity of the mold, glass, and nitrogen (J/(kg °C));

$m_{i}$—mass of the molds and glass blank (kg);

ΔT—temperature changes of the glass blank and molds (°C);

ΔT′—temperature changes of nitrogen (°C);

v—nitrogen flow rate (mL/s);

t—nitrogen injection time (s);

ρ—nitrogen density (kg/mm^3^).

**Figure 13:**
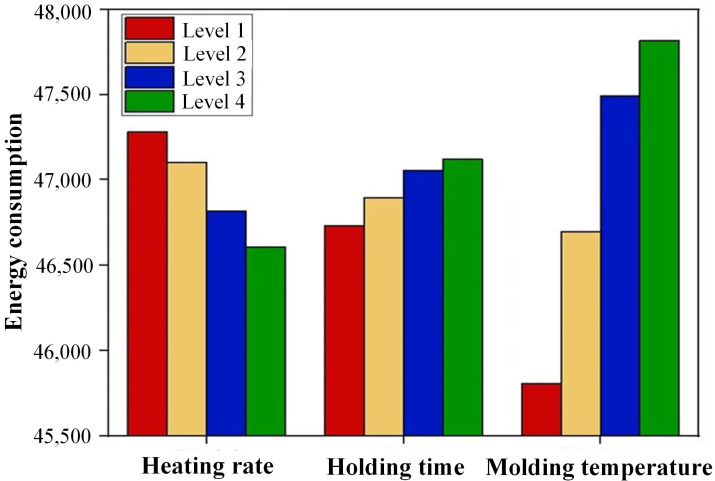
Influence of molding parameters on energy consumption.

Observing Table 9 reveals that the heating rate (*A*), holding time (*B*), and molding temperature (*C*) emerge as critical factors constraining the production efficiency of glass components. This trend is further illustrated in Figure 13, where a noticeable increase in molding temperature leads to a sharp rise in energy consumption. Simultaneously, extended holding times correspond to increased energy consumption. Conversely, faster heating rates yield lower energy consumption. Although elevating the molding temperature can mitigate dimensional deviations and stress levels, this increase in temperature directly leads to higher energy consumption during production. When heating rates decrease and heating times increase, the component’s production cycle lengthens, resulting in augmented furnace heat consumption and amplified energy consumption.

## 4. Optimization of Molding Process

### 4.1. Regression Model

Using a model-driven approach offers numerous advantages, but it is associated with relatively high simulation costs [39,40]. Artificial intelligence algorithms typically demand a significant volume of data for effective training and learning [41,42]. Insufficient data can potentially result in a deterioration of model performance. Therefore, a data-driven approach is favored for its ease of establishment. The relationship among the maximum residual stress, dimension deviation, and energy consumption of molded products is exceedingly complex and is far from a simple linear pattern, making the construction of an accurate analytical model a challenging task. To tackle this challenge, regression analysis was employed to incorporate various influencing factors into response statistics. This approach enables a more comprehensive depiction of the interactions among these factors, thus enhancing our understanding of their relationships. The model formula is presented as Equation (3):

(3) $$y(x)=a_{0}+\sum_{i=1}^{n}a_{i}x_{i}+\sum_{ij(i<j)}^{n}a_{ij}x_{i}x_{j}$$

where y is the objective function, $a_{0}$ is the zero-order coefficient, $a_{i}$ is the first-order coefficient, $a_{ij}$ is the second-order coefficient, $x_{i}$ and $x_{j}$ represent the variable factors, and n is the number of factors. By employing the commercial data analysis software Minitab 19, the regression models of the maximum residual stress ($R_{s}$), dimension deviation ($S_{d}$), and energy consumption ($E_{e}$) were established, as shown in Equations (4)–(6), respectively.

(4) $$\begin{array}{l} R_{s}=-2145+26.34A-1.0154B+8.315C-5.364D+81.70E-17.332A^{2}\\ -0.000695B^{2}-0.007894C^{2}+0.002025D^{2}-35.61E^{2}+0.02819AB+\\ 0.0875AC+0.0178AD+0.04197BD \end{array}$$

(5) $$\begin{array}{l} S_{d}=50-1.19A+0.0058B-0.173C+0.0132D-0.59E+0.068A^{2}+\\ 0.000001B^{2}+0.000151C^{2}+0.000047D^{2}+0.256E^{2}-0.00029AB+\\ 0.00147AC+0.00265AD-0.000176BD \end{array}$$

(6) $$\begin{array}{l} E_{e}=-411441+1914A-217B+1600C+113D-1136E-32A^{2}-\\ 0.0615B^{2}-1.415C^{2}-1.81D^{2}-619E^{2}-10.02AB-1.0AC-6.5AD\\ +0.463BD \end{array}$$

To evaluate the adequacy of the regression models in relation to the expected outcomes, an analysis of the coefficient of determination, *R*-sp, was conducted. The results are as follows: For the regression model of maximum residual stress ($R_{s}$), the *R*-sp was determined to be 0.9999, signifying a remarkably accurate model. In the case of the dimension deviation ($S_{d}$) regression model, the *R*-sp was calculated as 0.9610, with a minor deviation of 0.039, well within the acceptable error range for this model. Similarly, the energy consumption ($E_{e}$) regression model achieved an *R*-sp of 0.9985, attesting to its high level of precision.

### 4.2. Multi-Objective Optimization

The non-dominated sorting genetic algorithm II (NSGA-II) is an optimization method based on the NSGA algorithm, jointly proposed by Deb and Srinivas [43]. This algorithm is straightforward to implement and exhibits a faster optimization rate. NSGA-II is a multi-objective optimization technique that relies on Pareto optimality as its foundation and incorporates elite strategies. It rapidly and efficiently performs non-dominated sorting, thereby markedly enhancing the efficiency and precision of genetic algorithms [44]. It excels in optimizing structural parameters within models and demonstrates high precision in this regard. NSGA-II leverages crowding distance comparison operators and elite strategies to enhance optimization accuracy. The crowding distance comparison operator plays a vital role in assessing the density around each individual while selecting the new generation of individuals, ensuring that promising individuals are not discarded and promoting a more even distribution of non-dominated solutions across the entire solution space. In this study, the NSGA-II optimization algorithm finds application in the molding parameters for large-sized vehicle glass components. This not only reduces simulation computation time but also mitigates the risk of converging to local optima and encourages population diversity.

Utilizing NSGA-II, a multi-objective optimization of glass component molding parameters was conducted to reduce dimension deviation, residual stress, and energy consumption. The configuration of operational parameters was as follows:

(1) $f_{\min}=\left\{R_{s},S_{d},E_{e}\right\}$;
(2) Iteration number = 500;
(3) Population size = 100;
(4) Fitness function value deviation = 1 × 10^−100^;
(5) Crossover probability = 0.5;
(6) Mutation probability = 0.0005.

**Figure 14:**
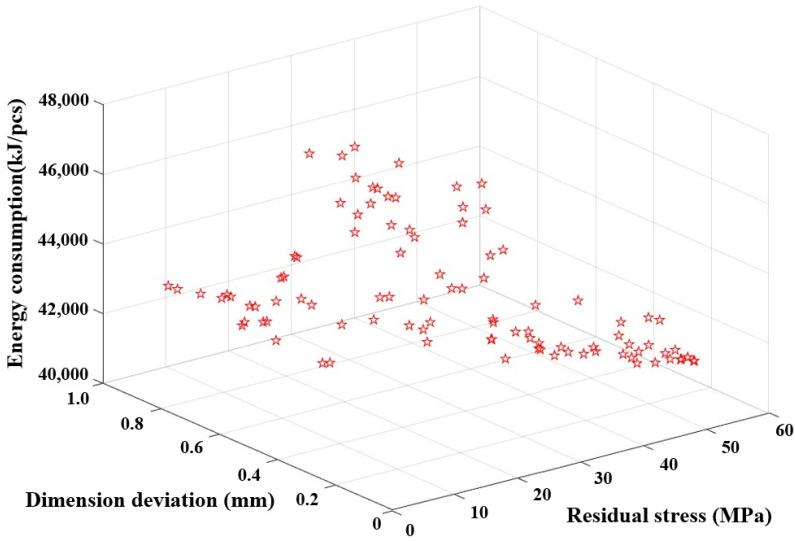
Pareto front solutions for residual stress, dimension deviation, and energy consumption.

Based on Figure 14, it is evident that achieving the optimal state for the dimension deviation, residual stress, and energy consumption simultaneously is not feasible. Therefore, in the implementation of multi-objective optimization, it is crucial to regulate the values of three objectives within acceptable ranges to achieve the best energy efficiency and reduce overall costs. Table 10 displays partial Pareto solutions for three optimization objectives. The optimal molding parameters are as follows: a heating rate of about 1.95 °C/s, a holding time of about 158 s, a molding temperature of about 570 °C, a molding pressure of about 34 MPa, and a cooling rate of about 1.15 °C/s. The optimized solutions successfully strike a balance between high quality and low energy consumption.

### 4.3. Experimental Validation

After optimization, the three-objective optimization partial Pareto front solutions could be obtained. For the validation of the Pareto front solutions concerning residual stress ($R_{s}$), dimension deviation ($S_{d}$), and energy consumption ($E_{e}$), a simulation and experimental verification were further conducted. Groups 3, 4, and 5 of the Pareto front solutions were selected to carry out numerical simulation studies. A comparison of the simulation results and these solutions facilitates a more precise evaluation of the optimized results.

**Figure 15:**
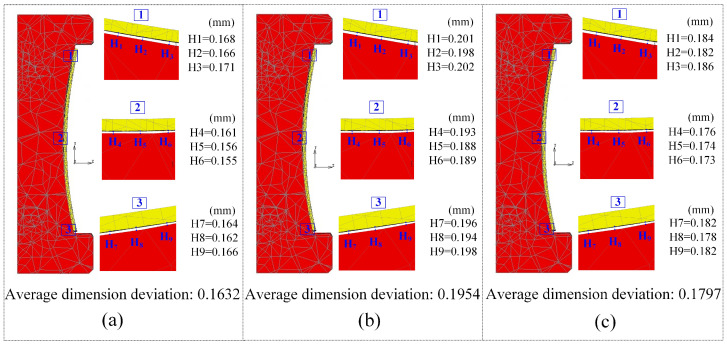
Dimension deviation of glass components: (**a**) Group 3; (**b**) Group 4; (**c**) Group 5.

**Figure 16:**
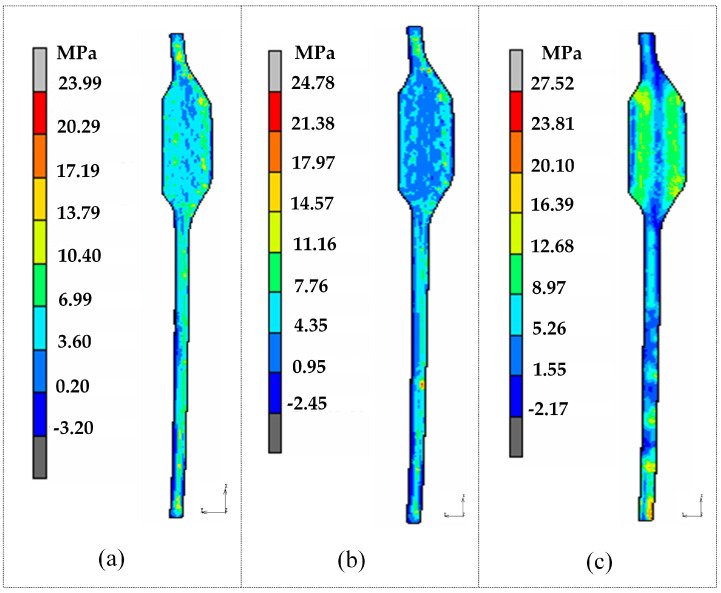
Maximum residual stress of glass components: (**a**) Group 3; (**b**) Group 4; (**c**) Group 5.

Figure 15 and Figure 16 display the results for dimension deviation and maximum stress. In the case of the third group scheme, the simulation results of the dimension deviation, maximum residual stress, and energy consumption are as follows: 0.1632 mm, 23.99 MPa, and 46,270.3 kJ/pcs, respectively. When comparing these results with the previous optimization results, it is evident that the relative error for maximum residual stress is 12.3%, for dimension deviation is 8.6%, and for energy consumption is 2.9%. Similarly, for the fourth group scheme, the relative errors between the simulation experiment results and the optimization results are 8.3%, 4.7%, and 7.7%, while for the fifth group scheme, they are 16.3%, 3.6%, and 7.1%, respectively. It can be observed that the optimized predictive results closely match the simulation results, with relative errors well controlled within a 20% margin. Detailed comparison results are shown in Table 11, all of which are within an acceptable range.

**Figure 17:**
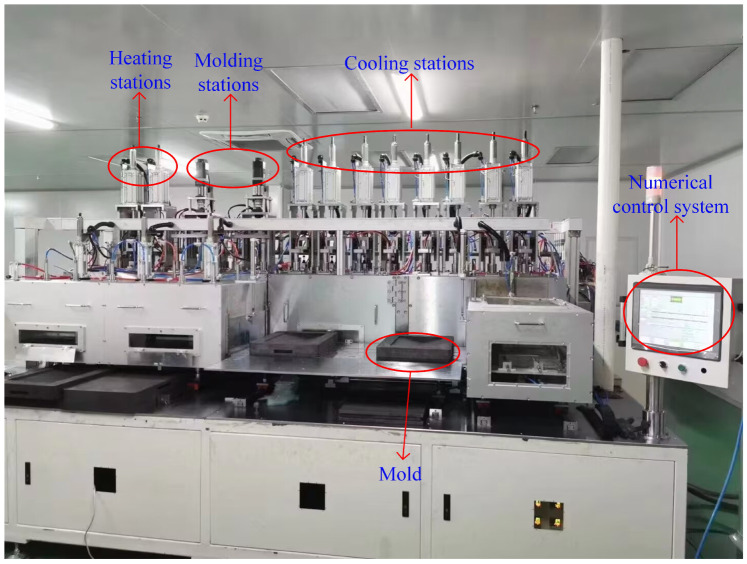
The device for the molding of large irregular glass components of vehicles.

**Figure 18:**
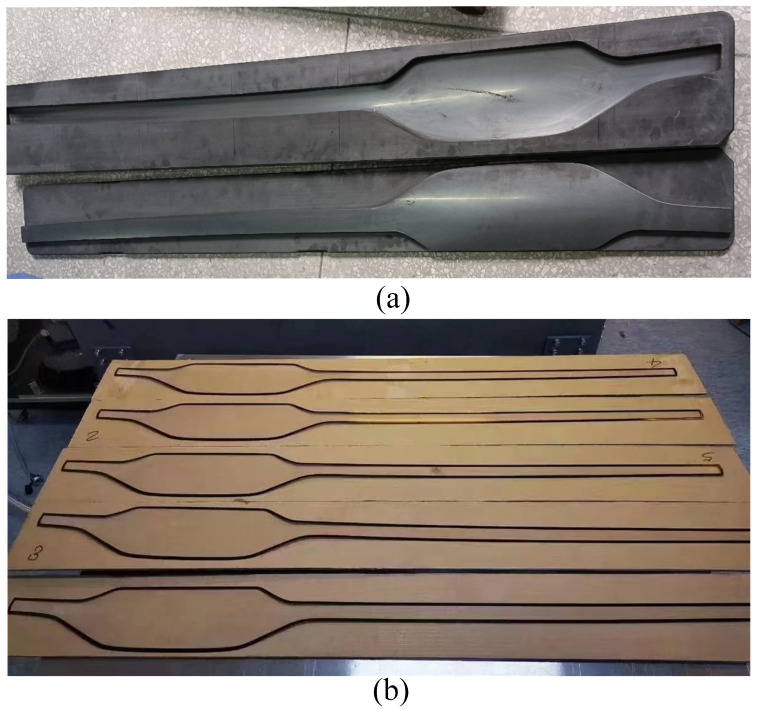
Large vehicle glass components and molds: (**a**) glass molds; (**b**) glass components.

The multifunctional molding machine shown in Figure 17, located at Guangdong Intelligent Robotics Research Institute, is equipped with a PLC automatic control system and a multifunctional assembly line, resulting in a substantial enhancement of production efficiency. The glass molding device includes a CNC system, heating stations (two stations), molding stations (two stations), cooling stations (eight stations), a furnace chamber, nitrogen supply and discharge devices, and auxiliary mechanisms like a power supply. The device’s dimensions are 25,850 × 4800 × 3600 mm, with a weight of 12,500 kg and a power rating of 400 KW. The CNC system employs programmable control technology and achieves precise closed-loop control through a feedback system incorporating high-resolution temperature sensors, pressure sensors, and gas flow sensors. During the heating stage, each station consists of six components: upper and lower heating plates (incorporating embedded heating tubes), upper and lower conductive plates, molds, and a glass sample. In the molding stage, hydraulic cylinders were employed to apply pressure to the molds. During the cooling stage, the heating elements progressively decreased in temperature. Figure 18 illustrates the mold and glass components.

Upon experimental verification, the average dimension deviation of the large-size vehicle glass components under the third scheme in Table 11 measures 0.1380 mm, which has an error of less than 20% compared to the simulation results. Precision molding of curved ultra-thin glass demands high molding dimension accuracy, typically controlling dimension deviation within the range of 10^−1^ mm. In conventional modeling processes, due to the limitations of numerical simulation modeling software, the existence of errors is difficult to avoid. For example, in the verification of an ultra-thin curved glass model, Yang et al. [45] found that the numerical results of the dimension deviation were generally lower than the experimental results, yet the variation trend between the simulation and experimental results remained consistent. He et al. [27] also obtained a maximum prediction error of 17.62% in the modeling of smartphone curved screen components. In addition, the glass component of a vehicle in this study possesses the characteristics of a large and irregular shape. The dimension deviation in the actual production is about ±0.15 mm, whereas the prediction error of 20% of the simulation model is at 10^−2^ mm. Therefore, taking into account potential experimental variations and the limitations of simulation modeling, the prediction error of less than 20% in this study falls within an acceptable range.

## 5. Conclusions

A molding model of an irregular glass component of a vehicle was established to predict and analyze the dominant factors of quality characteristics and energy consumption using a multi-variable factor experimental design. Subsequently, an optimization scheme was formulated to achieve a synergistic balance between quality characteristics and energy consumption, employing the NSGA-II algorithm. The main conclusions are as follows:

(1) The simulation model of large glass component molding was established, the temperature variation of the glass blank and graphite mold during the heating stage was analyzed, and the quality characteristics of the molded component were precisely predicted. The results indicate that the stress is predominantly concentrated in the bending deformation position of the molded component, with the maximum dimension deviation occurring at the central position.
(2) Among the various factors, the molding temperature, molding pressure, and cooling rate have the most significant impact on the molding process of glass components. Under the combination of a molding temperature of 580 °C, molding pressure of 25 MPa, and cooling rate of 1.25 °C/s, the residual stress remains consistently low. Similarly, under the combination of a molding temperature of 570 °C, molding pressure of 30 MPa, and cooling rate of 1.25 °C/s, the dimension deviation is kept to a minimum. Furthermore, with a molding temperature of 550 °C, molding pressure of 30 MPa, and cooling rate of 1 °C/s, lower energy consumption in the production of glass components can be obtained.
(3) The combination of a heating rate of 1.95 °C/s, holding time of 158 s, molding temperature of 570 °C, molding pressure of 34 MPa, and cooling rate of 1.15 °C/s was determined as the cooperative balance scheme for quality characteristics and energy consumption by NSGA-II there-objective optimization. The optimized prediction closely aligned with both simulation and experimental results, with a maximum error not exceeding 20%, well within the acceptable range.

## Figures and Tables

**Table 1:** Dimensions of molded glass components.

Glass Components	Dimension	Aspect Ratio	Irregular
Vehicle glass component	1454 × 161 × 2 mm	9.03	Yes
Smartphone 3D curved screen [27]	148 × 73 × 0.35 mm	2.03	No
Fingerprint lock glass panel [30]	348 × 66.6 × 2 mm	5.23	No
Mobile display obtuse glass component [31]	*Φ* = 14 mm; *h* = 7 mm	1	No
Alvarez lens [32]	*Φ* = 84 mm; *h* = 1 mm	1	Yes
Aspherical glass component [33]	13 × 3.5 × 1 mm	3.71	No

**Table 2:** Mechanical and thermal properties of glass and mold materials.

Properties	Glass Material	Mold Material
Density *ρ* (g/cm^3^)	2.39	1.78
Young’s modulus *E* (GPa)	77	10.2
Poisson rate *ν*	0.22	0.25
Thermal conductivity *K* (W/m·°C)	1.028	151
Specific heat *C*_p_ (J/kg·°C)	858	720
Thermal expansion coefficient (/°C)	7.25 × 10^−6^	4.8 × 10^−6^

**Table 3:** Viscosity of third-generation Corning glass at different temperatures.

No.	Temperature (°C)	Viscosity
1	574	10^14.7^
2	628	10^13.2^
3	900	10^7.60^

**Table 5:** Control factors and standard settings of levels.

No.	Control Factors				
	*A* (°C)	*B* (°C/s)	*C* (s)	*D* (MPa)	*E* (°C/s)
1	1.5	100	550	20	0.75
2	2.0	120	560	25	1
3	2.5	140	570	30	1.25
4	3.0	160	580	35	1.5

**Table 6:** Experimental design and response statistics.

No.	Control Factors					Residual Stress (MPa)	Dimension Deviation (mm)	Energy Consumption (kJ/pcs)
	*A*	*B*	*C*	*D*	*E*			
1	1.5	100	550	20	0.75	28.98	0.2537	45,921.5
2	1.5	120	560	25	1	23.16	0.2586	46,942.6
3	1.5	140	570	30	1.25	19.92	0.1851	47,937.1
4	1.5	160	580	35	1.5	18.69	0.2307	48,328.3
5	2	100	560	30	1.5	24.87	0.1913	46,573.2
6	2	120	550	35	1.25	26.78	0.2014	45,977.2
7	2	140	580	20	1	16.69	0.2374	47,981.6
8	2	160	570	25	0.75	19.07	0.2713	47,879.3
9	2.5	100	570	35	1	20.07	0.1928	47,113.4
10	2.5	120	580	30	0.75	17.97	0.2377	47,631.1
11	2.5	140	550	25	1.5	30.06	0.2456	45,777.9
12	2.5	160	560	20	1.25	22.32	0.2230	46,737.3
13	3	100	580	25	1.25	17.55	0.2310	47,318.8
14	3	120	570	20	1.5	18.72	0.2020	47,032.3
15	3	140	560	35	0.75	26.16	0.2210	46,524.7
16	3	160	550	30	1	31.27	0.2053	45,539.2

**Table 7:** Mean response table for residual stress.

Level	*A*	*B*	*C*	*D*	*E*
1	22.69	22.87	31.02	21.28	23.04
2	23.21	23.41	24.13	22.46	22.40
3	22.60	22.81	19.45	23.51	23.39
4	23.42	22.84	17.33	24.68	23.09
Delta	0.82	0.60	13.70	3.40	1.00
Order	4	5	1	2	3

**Table 8:** Mean response table for dimension deviation.

Level	*A*	*B*	*C*	*D*	*E*
1	0.2320	0.2172	0.2265	0.2290	0.2459
2	0.2253	0.2249	0.2235	0.2516	0.2235
3	0.2248	0.2223	0.2128	0.2049	0.2101
4	0.2148	0.2326	0.2342	0.2115	0.2174
Delta	0.0172	0.0154	0.0214	0.0468	0.0358
Order	4	5	3	1	2

**Table 9:** Mean response table for energy consumption.

Level	*A*	*B*	*C*	*D*	*E*
1	47,282	46,732	45,804	46,918	46,989
2	47,103	46,896	46,694	46,980	46,894
3	46,815	47,055	47,491	46,920	46,993
4	46,604	47,121	47,815	46,986	46,928
Delta	679	389	2011	68	98
Order	2	3	1	5	4

**Table 10:** Partial Pareto front solutions for three-objective optimization.

No.	Control Factors					Residual Stress (MPa)	Dimension Deviation (mm)	Energy Consumption (kJ/pcs)
	*A*	*B*	*C*	*D*	*E*			
1	1.873	158.34	572.47	33.861	1.239	19.71	0.1903	44,770
2	1.906	158.41	567.47	34.365	1.034	20.60	0.1966	45,280
3	1.997	158.30	574.55	34.144	1.147	21.04	0.1772	44,920
4	1.844	154.18	566.93	34.650	1.109	22.73	0.1863	44,240
5	2.069	159.26	571.12	34.959	1.139	23.04	0.1862	45,180
6	2.108	158.34	573.75	34.729	1.181	23.48	0.1868	44,800
7	1.918	157.04	567.47	34.512	1.068	23.80	0.1832	45,600
8	2.125	158.54	572.31	34.811	1.252	24.21	0.1832	44,590

**Table 11:** Simulation validation of optimization results.

No.	Control Factors					Simulation Results			Relative Error		
	*A* (°C/s)	*B* (s)	*C* (°C)	*D* (MPa)	*E* (°C/s)	*R_s_* (MPa)	*S_d_* (mm)	*E_e_* (kJ/pcs)	*R_s_* (%)	*S_d_* (%)	*E_e_* (%)
3	1.997	158.30	574.55	34.144	1.147	23.99	0.1632	46,270.3	12.3	8.6	2.9
4	1.844	154.18	566.93	34.650	1.109	24.78	0.1954	47,951.2	8.3	4.7	7.7
5	2.069	149.26	571.12	34.959	1.139	27.52	0.1797	48,651.2	16.3	3.6	7.1

## Data Availability

Not applicable.
